# NSMCE2 dispensability in mouse spermatogenesis suggests functional redundancy within the meiotic repair network

**DOI:** 10.3389/fcell.2026.1751806

**Published:** 2026-03-03

**Authors:** Zihan Qin, Qiaohua Xiong, Mei Wang, Yuchen Fang, Qigang Fan, Binyu Ma, Ying Gao, Muyang Cheng, Yuming Cao, Yuanzhen Zhang

**Affiliations:** 1 Center for Reproductive Medicine, Zhongnan Hospital of Wuhan University, Wuhan, Hubei, China; 2 Clinical Medicine Research Center of Prenatal Diagnosis and Birth Health in Hubei Province, Wuhan, Hubei, China; 3 Wuhan Clinical Research Center for Reproductive Science and Birth Health, Wuhan, Hubei, China; 4 Department of Urology, Zhongnan Hospital of Wuhan University, Wuhan, Hubei, China; 5 Department of Obstetrics and Gynecology, Tongji Hospital, Tongji Medical College, Huazhong University of Science and Technology, Wuhan, Hubei, China

**Keywords:** functional redundancy, male fertility, meiosis, NSMCE2, spermatogenesis

## Abstract

NSMCE2, a SUMO E3 ligase subunit of the SMC5/6 complex, is essential for maintaining genomic integrity during mitosis, yet its meiotic function remains poorly understood. Here, we investigated the physiological role of NSMCE2 in male germ cells using a conditional knockout mouse model. Despite its high and stage-specific expression in testes, germline deletion of *Nsmce2* resulted in no apparent impairment of spermatogenesis or fertility. Comprehensive analyses revealed normal testicular architecture, unaltered meiotic progression, intact DNA double-strand break repair, and stable transcriptomic profiles in *Nsmce2*-deficient testes. Mechanistically, we observed a specific upregulation of the SUMO E3 ligase PIAS1, which suggests a potential role in maintaining global SUMOylation homeostasis in the deficiency of NSMCE2. These findings demonstrate that NSMCE2 is dispensable for male fertility under physiological conditions. Such dispensability suggests that the meiotic DNA repair network possesses substantial functional redundancy and robust compensatory capacity, ensuring the maintenance of spermatogenic integrity even in the absence of a key repair factor. Collectively, our study highlights the resilient and fail-safe design of the mammalian meiotic system, which safeguards fertility through intrinsic robustness and molecular redundancy.

## Graphical Abstract

**Figure:**
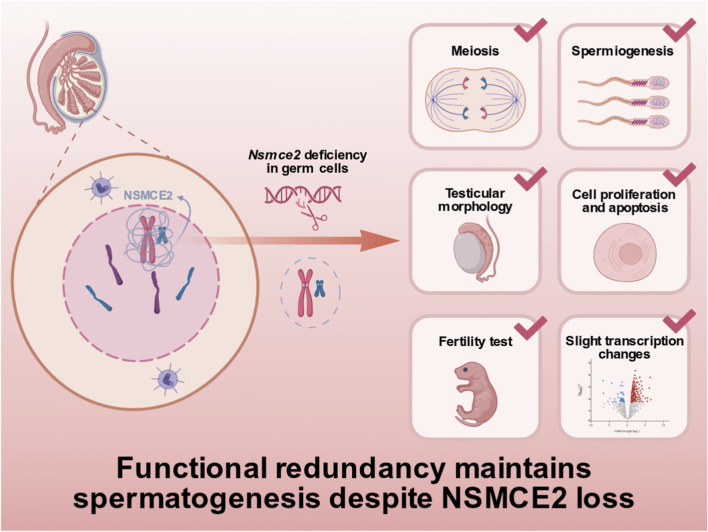

## Introduction

Spermatogenesis is a complex and tightly regulated process that ensures the continuous production of male gametes (Barada et al., 2025; Dong et al., 2022; Wang et al., 2022). Meiosis represents the most critical phase of this process, as it generates haploid spermatids and contributes to genetic diversity through homologous chromosome segregation and recombination (Ito and Shinohara, 2022; Zickler and Kleckner, 2023). The accurate repair of programmed DNA double-strand breaks (DSBs) during meiotic prophase I is essential for successful homologous recombination and chromosomal integrity (Lukaszewicz et al., 2021; Sole et al., 2023). Failure in this repair leads to meiotic arrest, apoptosis, and male infertility (Jiang et al., 2023; Wu et al., 2021; Xu et al., 2023; Yin et al., 2025). To accomplish this challenging task, germ cells rely on a series of DNA damage response (DDR) factors and chromosome structural proteins that together ensure the fidelity of meiotic progression (Bolanos-Villegas, 2021; Gao et al., 2025; Nie et al., 2024).

Among these regulatory complexes, the Structural Maintenance of Chromosomes (SMC) 5/6 complex acts as a crucial guardian of genomic integrity. It facilitates homologous recombination, prevents chromosome entanglement, and preserves genomic stability during both mitosis and meiosis (Aragon, 2018; Raschle et al., 2015; Sole-Soler and Torres-Rosell, 2020). As a component of SMC5/6, non-SMC element 2 (NSMCE2, also known as MMS21) provides SUMO E3 ligase activity through its SP-RING domain, and has been shown to promote DNA repair and replication fork stability in somatic cells (Potts et al., 2006; Potts and Yu, 2005; Wu et al., 2012). The embryonic lethality observed in *Nsmce2* full knockout mice highlights its indispensable role in early development and somatic genome maintenance (Jacome et al., 2015). However, its physiological function in the germline, where chromosomal events are far more dynamic, remains largely undefined.

Given the elevated expression of NSMCE2 in testes and its specific localization to meiotic chromatin regions such as the XY body, it was reasonable to hypothesize that NSMCE2 is essential for maintaining meiotic genome integrity and thus male fertility. Surprisingly, our findings demonstrate that the loss of NSMCE2 in germ cells does not result in discernible defects in meiosis or fertility. This unexpected observation provides an opportunity to explore how the meiotic system sustains stability despite the absence of a canonical DNA repair enzyme.

We propose that this phenomenon reflects an inherent property of the spermatogenic program—its systemic robustness and functional redundancy. During evolution, the germline has likely developed overlapping and compensatory mechanisms to guarantee reproductive continuity across generations, ensuring that the failure of a single component does not compromise fertility. In this study, we generated a germline-specific *Nsmce2* knockout mouse model to interrogate the role of NSMCE2 during meiosis. By integrating histological, cytological, and transcriptomic analyses, we uncovered that spermatogenesis proceeds normally in the absence of NSMCE2, revealing a resilient and redundant architecture within the meiotic DNA repair network. These findings highlight how the male germline embodies a robust biological system optimized to preserve genetic integrity even under molecular perturbations.

## Materials and methods

### Animals and ethics statement

All animal experiments were conducted in compliance with the ethical standards of the Institutional Animal Care and Use Committee (IACUC) in Zhongnan Hospital of Wuhan University (approval number: ZN2025039). Mice were maintained under specific pathogen-free (SPF) conditions with a 12 h light/dark cycle and *ad libitum* access to food and water. All procedures were performed in accordance with the guidelines of the National Institutes of Health for the Care and Use of Laboratory Animals.

### Generation and genotyping of *Nsmce2* cKO mice

To investigate the physiological function of NSMCE2 in male germ cells, we generated a germ cell–specific *Nsmce2* conditional knockout (cKO) mouse model based on the canonical *Nsmce2* transcript (RefSeq: NM_001374768.1). The *Nsmce2* ^*flox/flox*^ line was established by inserting two loxP sites flanking exon 3 of the *Nsmce2* gene (Cyagen Biosciences, China). These mice were crossed with *Stra8-Cre* transgenic mice, in which Cre recombinase is expressed in pre-meiotic spermatogonia starting from 3 days postpartum (3 dpp) (Li et al., 2024). Offspring with the genotype *Nsmce2* ^*flox/-*^ *; Stra8-*Cre were designated as cKO mice, while *Nsmce2* ^*flox/-*^ littermates were used as controls. Genotyping was performed by polymerase chain reaction (PCR) using tail DNA and specific primers: *Nsmce2*-F1: 5′-ACG​GTA​GTT​GAG​CTT​TCT​TCT​GTA​T-3′, *Nsmce2*-R1: 5′-AGC​ACC​ATA​TCT​GGC​TAA​GTG​AG-3′, *Nsmce2*-F2: 5′-CCTCTTTA GTA​GTG​CCA​TGT​TGA​G-3′, *Nsmce2*-R2: 5′-ATA​AAG​AAA​CCA​GAA​GTG​CAA​GGCA-3′.

### Fertility assessment

To evaluate reproductive performance, 8 week-old cKO and WT male mice were each paired with two adult WT females for at least six consecutive months. The number of litters and the total number of pups sired by each male were recorded. To assess fertility capacity, the average litter size and the cumulative number of offspring per female were calculated and statistically analyzed.

### Histological analysis

Testes and epididymides from adult mice were collected, fixed in Bouin’s solution, embedded in paraffin, and sectioned at 5 μm thickness. Slides were stained with hematoxylin and eosin (H&E) following standard protocols. The morphology of seminiferous tubules, germ cell organization, and epididymal sperm content were examined using a Nikon light microscope (Tokyo, Japan). At least 50 seminiferous tubules per animal were evaluated to ensure reproducibility.

### Immunofluorescence staining

For immunofluorescence, testes were fixed in 4% paraformaldehyde, dehydrated in 30% sucrose, embedded in OCT compound, and cryosectioned at 7 μm. After antigen retrieval and blocking in 5% bovine serum albumin (BSA), sections were incubated overnight at 4 °C with primary antibodies, followed by fluorophore-conjugated secondary antibodies. The following primary antibodies were used: anti-NSMCE2 (1:100, Proteintech, 13627-1-AP), anti-DDX4 (1:200, Abcam, ab13840), anti-SOX9 (1:500, Abclonal, A19710), anti-PCNA (1:200, Proteintech, 10205-2-AP), anti-γH2AX (1:200, Solarbio, K001451M). After counterstaining with DAPI (Sigma-Aldrich), sections were mounted and imaged using a confocal laser scanning microscope (Zeiss LSM880, Germany). Co-localization analyses were performed using ImageJ software (NIH).

### Western blotting

Total protein extracts were obtained from testes and other tissues using RIPA buffer (Beyotime Biotechnology) supplemented with PMSF protease inhibitor. After homogenization and centrifugation at 12,000 rpm for 10 min at 4 °C, the supernatants were mixed with SDS loading buffer, boiled for 10 min, and subjected to SDS-PAGE. Proteins were transferred onto PVDF membranes (Millipore) and blocked in 5% non-fat milk in TBST for 1 h, and incubated overnight at 4 °C with the primary antibody. After three washes in TBST, the membranes were incubated with HRP-conjugated secondary antibody (Proteintech) at room temperature for 1 h. The primary antibodies used were as follows: anti-GAPDH (1:100000, Proteintech, 60004-1-Ig), anti-βTubulin (1:2500, Proteintech, 10094-1-AP), anti-NSMCE2 (1:1000, Proteintech, 13627-1-AP), anti-SUMO1 (1:1000, Abclonal, A19121), anti-SUMO2/3 (1:500, Abclonal, A5066), anti-PIAS1 (1:3000, Proteintech, 23395-1-AP), anti-PIAS4 (1:500, Proteintech, 14242-1-AP), anti-ZNF451 (1:500, Proteintech, 25228-1-AP).

### Sperm collection and analysis

Cauda epididymides were dissected from 8 week-old mice and minced in 1 mL of phosphate-buffered saline (PBS), followed by incubation at 37 °C for 30 min to release sperm. Sperm were stained using Giemsa solution (Servicebio, China) and analyzed under a light microscope. Sperm concentration and motility were quantified using a hemocytometer, and morphological abnormalities were evaluated in at least 200 sperm per sample.

### Quantitative reverse-transcription PCR

Total RNA from tissues was extracted with TRIzol reagent, and then reverse transcribed with HiScript® II Q RT SuperMix for qPCR (+gDNA wiper) kit (Vazyme), and amplified with ChamQ SYBR qPCR Master Mix kit (Vazyme). The expression level was normalized to *Gapdh* expression. The primer sequence used was as follows: *Gapdh*-F: 5′-AGG​TCG​GTG​TGA​ACG​GAT​TTG-3′, *Gapdh*-R: 5′-TGTAGACCATGTAGTTGAGGTCA-3′, *Nsmce2*-F: 5′-GTG​GGA​CAG​CCA​GGT​AAC​AA-3′, *Nsmce2*-R: 5′-AGC​AAA​CTC​AAC​CAT​GGC​CT-3′.

### Spermatocyte spreading and immunostaining

Spermatocyte nuclear spreads were prepared as described previously (Fang et al., 2021). Briefly, seminiferous tubules were incubated in hypotonic extraction buffer (30 mM Tris, 50 mM sucrose, 17 mM trisodium citrate, 5 mM EDTA, pH 8.2) for 30 min and gently dispersed. Cell suspensions were dropped onto slides coated with 1% paraformaldehyde containing 0.15% Triton X-100 and air-dried overnight. For immunostaining, slides were blocked with 5% BSA and incubated with primary antibodies against SYCP3, SYCP1, γH2AX, SUMO1, MDC1, and RNA pol II at 4 °C overnight, followed by Alexa Fluor–conjugated secondary antibodies (Invitrogen). Nuclei were counterstained with DAPI and mounted with antifade reagent. Images were captured using a Zeiss LSM880 confocal microscope, and at least 100 pachytene spermatocytes per animal were analyzed for synapsis and recombination foci using ImageJ software. The primary antibodies used were as follows: anti-γH2AX (1:200, Solarbio, K001451M), anti-SYCP1 (1:200, Abcam, ab15090), anti-SYCP3 (1:200, Abcam, ab15093), anti-SUMO1 (1:100, Abclonal, A19121), anti-MDC1 (1:50, Abclonal, A12714), anti-RNA pol II (1:50, Abcam, ab193468).

### RNA sequencing and analysis

Testis samples were collected from 8 week-old testes, followed by RNA extraction, RNA detection, library construction, sequencing, and data analysis. Differentially expressed genes were filtered using the criteria |log2FoldChange| ≥ 1 and FDR <0.05. Above processes were performed by Metware Company.

### Statistical analysis

All experiments were independently repeated at least three times using biological replicates. Data calculations and graphing were performed using GraphPad Prism 9. Data were expressed as mean ± standard error of the mean (SEM). Statistical analysis was conducted using Student’s t-test. P values <0.05 were considered statistically significant.

## Results

### NSMCE2 is enriched in mouse testes and its expression is developmentally regulated

**FIGURE 1:**
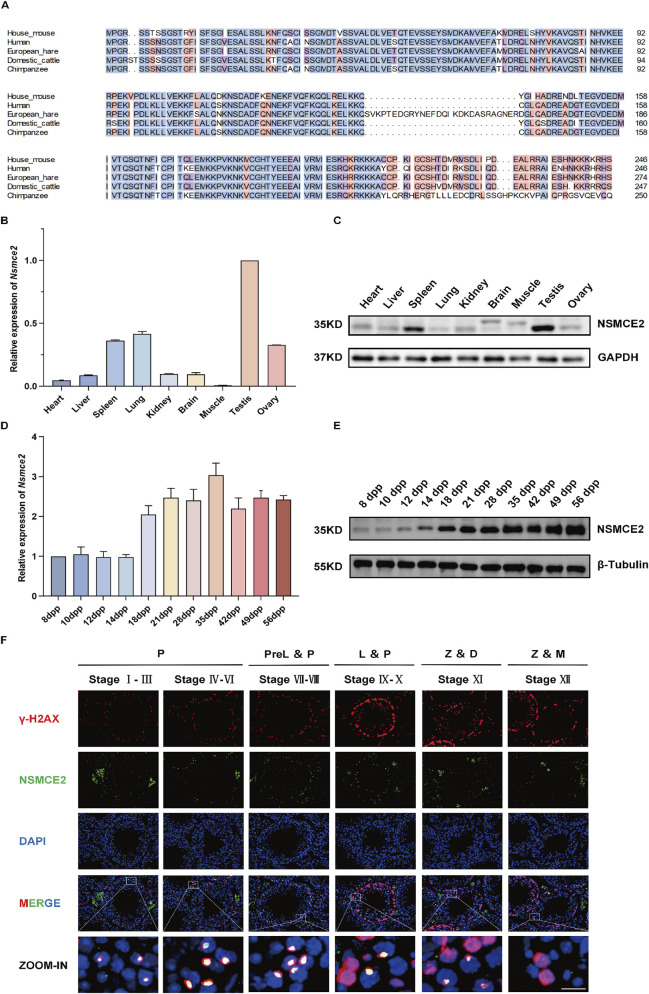
NSMCE2 is an evolutionarily conserved and testis-enriched protein whose expression is developmentally regulated **(A)** Multiple sequence alignment of NSMCE2 protein among specified species **(B)** Quantitative PCR analysis of *Nsmce2* mRNA expression in various tissues of 8-week-old mice (n = 3). Data were normalized using *Gapdh* as an internal reference. Data were normalized to testis expression as 1 **(C)** Western blotting analysis of NSMCE2 protein expression in various tissues of 8-week-old mice (n = 3). Data were normalized using GAPDH as an internal reference **(D)** Quantitative PCR analysis of *Nsmce2* mRNA expression in testes from mice of different ages (n = 3). Data were normalized using *Gapdh* as an internal reference. Data were normalized to expression of *Nsmce2* at 8 dpp as 1 **(E)** Western blotting analysis of NSMCE2 protein expression in testes from mice of different ages (n = 3). Data were normalized using β-Tubulin as an internal control **(F)** Testicular sections from 8-week-old wild-type mice were co-stained with γ-H2AX (red), NSMCE2 (green), and DAPI (blue). Scale bar: 50 μm (main image); 20 μm (zoomed-in image). P, pachytene; PreL, preleptotene; L, leptotene; Z, zygotene; D, diplotene; M, metaphase.

Multiple sequence alignment revealed that NSMCE2 is highly conserved across species. Specifically, the sequence similarity between human and mouse is as high as 84.21%, which indicates the conservation of biological function (Figure 1A). To investigate the expression pattern of *Nsmce2*, we validated its expression in various tissues of adult mice using quantitative reverse-transcription PCR (qRT-PCR) and Western blotting. We found that *Nsmce2* expression, both at the mRNA and protein levels, was significantly higher in the testes than in other organs such as the heart, brain, and lung (Figures 1B,C).

Furthermore, we investigated changes in *Nsmce2* expression across different postnatal testicular stages (Figures 1D,E). From postpartum day 8 (8dpp) to 14dpp, both mRNA and protein levels of NSMCE2 remained low and relatively stable. A striking increase was observed at 18dpp. Subsequently, from 21dpp to 56dpp, NSMCE2 expression remained relatively stable at high levels. The sharp upregulation of NSMCE2 at 18dpp coincides with a prominent phase within the first wave of meiosis, a period during which spermatocytes are predominantly in the pachytene and diplotene stages. This suggests that NSMCE2 may play a potential role in these specific meiotic phases. Consistently, single-cell sequencing data from the FertilityOnline database (FertilityOnline, 2021) indicate that NSMCE2 exhibits higher expression during the meiotic stages than in spermatids and somatic cells in the testis of adult mice. By co-staining NSMCE2 with γ-H2AX (a DSB marker) and DAPI in testicular immunofluorescence, it was found that NSMCE2 was specifically expressed in the nuclei of pachytene and diplotene spermatocytes (Figure 1F; Supplementary Figure S1).

### Generation and verification of germ cell–specific *Nsmce2* knockout mice

**FIGURE 2:**
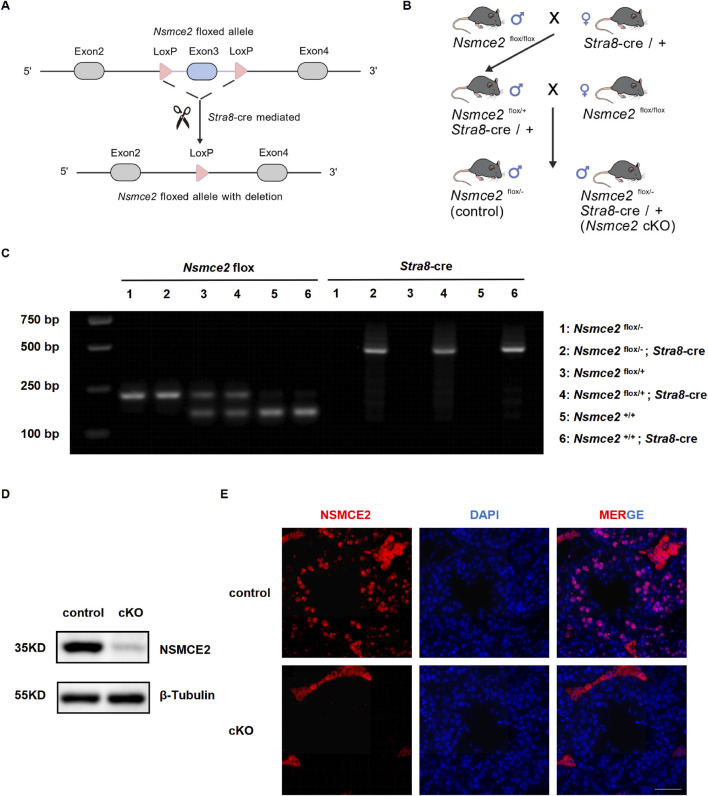
Construction and validation of germ cell-specific *Nsmce2* conditional knockout mice **(A)** Schematic diagram of the conditional knockout strategy for the *Nsmce2* Allele **(B)** Schematic Diagram of breeding strategy for germ cell-specific *Nsmce2* conditional knockout mice **(C)** Genotyping analysis of mouse tail genomic DNA using primers F2/R2. PCR products for *Nsmce2* alleles (floxed: 213 bp, WT: 146 bp) and the *Stra8*-Cre transgene were resolved by agarose gel electrophoresis. The numbered lanes (1–6) correspond to individual mice with the following genotypes: 1: *Nsmce2* ^*flox/-*^, 2: *Nsmce2* ^*flox/-*^; *Stra8*-cre, 3: *Nsmce2* ^flox/+^, 4: *Nsmce2* ^*flox/+*^; *Stra8*-cre, 5: *Nsmce2* ^*+/+*^, 6: *Nsmce2* ^+/+^; *Stra8*-cre. The deletion allele is not amplified by the *Nsmce2* genotyping primers due to the excision of the forward primer binding site **(D)** Western blotting analysis of NSMCE2 expression in testes from 8-week-old control and cKO mice (n = 3). Data were normalized using β-Tubulin as an internal control **(E)** Testicular sections from 8-week-old control and cKO mice (n = 3) were co-stained with NSMCE2 (red) and DAPI (blue). Scale bar: 50 μm.

To investigate the physiological role of NSMCE2 in spermatogenesis, we generated germ cell–specific *Nsmce2* conditional knockout (cKO) mice. Using CRISPR-Cas9 technology, we designed a strategy to target exon 3 of the *Nsmce2* gene on chromosome 15 in testicular germ cells, aiming to generate a 1750 bp gene fragment deletion. This deletion is predicted to lead to the loss of *Nsmce2* gene function in mice (Figure 2A). Specifically, *Nsmce2* ^*flox/flox*^ mice were generated by inserting a LoxP site on both sides of exon 3. Crossbreeding *Nsmce2* ^*flox/flox*^ mice with *Stra8*-Cre mice yielded *Nsmce2* ^*flox/-*^; *Stra8*-Cre mice (hereafter termed *Nsmce2*-cKO) and *Nsmce2* ^*flox/-*^ mice (hereafter termed control) after two generations (Figure 2B).

Following polymerase chain reaction (PCR) amplification of genomic DNA extracted from mouse tails, genotypes were identified via agarose gel electrophoresis. *Nsmce2* ^*flox/-*^ mice exhibited only the floxed allele band (213 bp), and wildtype (WT) mice showed only the WT allele band (146 bp), while *Nsmce2* ^*flox/+*^ mice displayed both the floxed allele band (213 bp) and the WT allele band (146 bp) (Figure 2C). Furthermore, RT-PCR analysis of testicular cDNA confirmed the presence of the specific deletion allele (about 300–400 bp) in cKO mice, verifying the successful excision of exon 3 at the transcript level (Supplementary Figure S2). Western blotting and immunofluorescence also confirmed the successful knockout of NSMCE2 in germ cells (Figures 2D,E). These results confirmed the successful generation of germline-specific *Nsmce2* knockout mice.

### *Nsmce2* deficiency in germ cells does not affect testicular development or fertility

**FIGURE 3:**
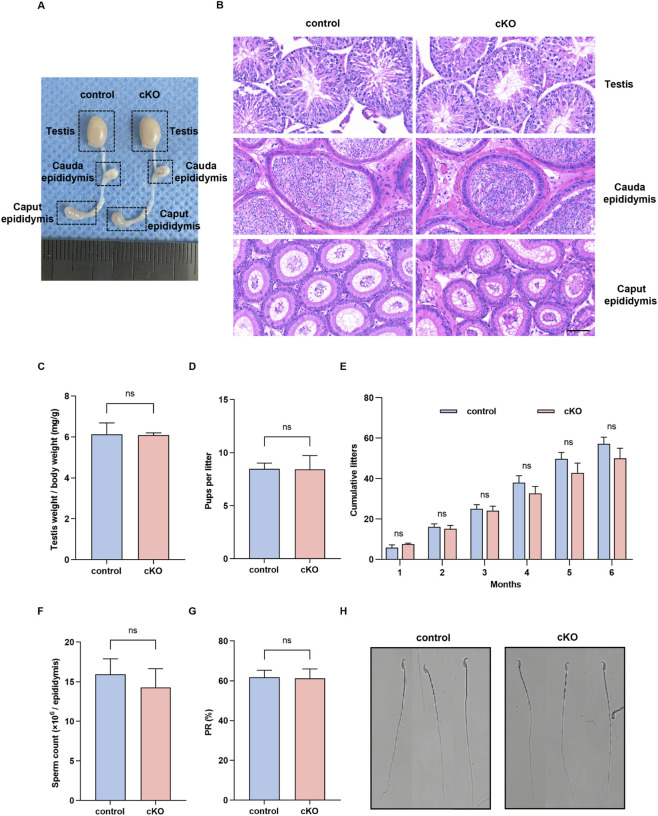
*Nsmce2* deficiency in germ cells does not compromise gross male reproductive phenotypes **(A)** Gross morphology of testes and epididymides in 8-week-old control and cKO mice **(B)** H&E staining of testes, cauda epididymis, and caput epididymis in 8-week-old control and cKO mice. Scale bar: 60 μm **(C)** Quantitative analysis of the testis-to-body weight ratio in 8-week-old control and cKO mice (n = 3). ns, not significant **(D)** Average size of litters sired by control and cKO male mice (n = 5). ns, not significant **(E)** Cumulative number of offspring sired by control and cKO male mice (n = 5) **(F)** Sperm counts in 8‐week‐old control and cKO mice (n = 4). ns, not significant **(G)** Percentage of forward-moving sperm in 8-week-old control and cKO mice (n = 4). ns, not significant **(H)** Giemsa staining of sperm from 8-week-old control and cKO mice.

To assess the impact of *Nsmce2* deficiency on male fertility, we conducted a comprehensive analysis of reproductive organ morphology and function. Macroscopic examination revealed no discernible differences in testicular and epididymal size or morphology between *Nsmce2* cKO mice and control mice (Figure 3A). Consistent with this, hematoxylin-eosin (H&E) staining revealed normal seminiferous tubule architecture in cKO mouse testes, with comparable sperm content in both the caput and cauda epididymides compared to controls (Figure 3B). The testis-to-body weight ratio remained unchanged in cKO mice (Figure 3C).

Crucially, functional fertility assessments revealed that *Nsmce2* cKO male mice exhibited full reproductive capacity. Their average litter size (Figure 3D) and cumulative number of offspring produced within 6 months post-mating (Figure 3E) showed no statistically significant differences compared to controls. Furthermore, sperm analysis revealed no significant defects in key parameters, including sperm count (Figure 3F), percentage of forward-moving sperm (Figure 3G), and sperm with normal morphology (Figure 3H). Collectively, these data indicate that germ cell-specific knockout of *Nsmce2* does not result in apparent impairment of testis development, spermatogenesis, or male fertility.

### Deficiency of *Nsmce2* in germ cells has little effect on their cellular homeostasis

**FIGURE 4:**
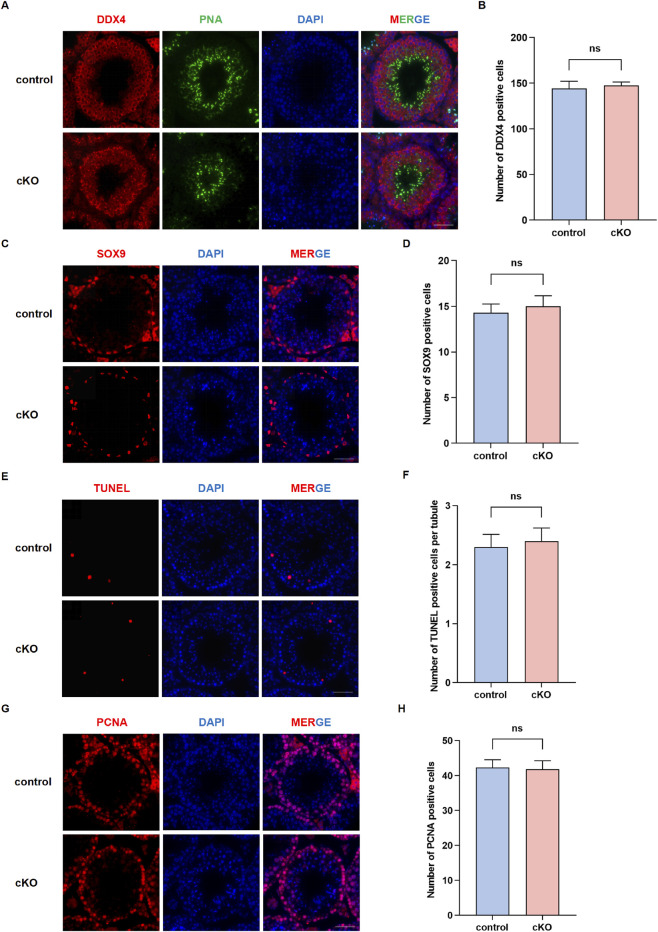
Deficiency of *Nsmce2* in germ cells has little effect on their cellular homeostasis. Immunofluorescence staining of testicular sections from 8-week-old control and cKO mice: DDX4 and PNA **(A)**, SOX9 **(C)**, TUNEL **(E)**, PCNA **(G)**. Scale bar: 50 μm. Statistical analysis of immunofluorescently positive cells in each tubule: DDX4 **(B)**, SOX9 **(D)**, TUNEL **(F)**, PCNA **(H)**. Data represent n = 10 biologically independent mice per group, with 100 seminiferous tubules analyzed per mouse. ns, not significant.

To investigate whether NSMCE2 deficiency disrupts testicular cellular architecture or induces cellular stress responses, we performed immunofluorescence staining for key molecular markers. Immunofluorescence staining for DDX4 (a pan-germ cell marker) confirmed the presence of germ cells at all stages within the seminiferous tubules of *Nsmce2* cKO mice, with distribution and density comparable to controls (Figures 4A,B). PNA staining (marking the acrosome of developing spermatids) revealed normal patterns of acrosome formation and elongation in spermatids at specific stages (Figure 4A). Furthermore, SOX9 staining (a marker specific to Sertoli cells) showed no alteration in the number or distribution of Sertoli cells in cKO testes (Figures 4C,D).

To directly assess potential cellular damage, we performed TUNEL staining to detect apoptosis. Results showed no significant increase in TUNEL-positive cells per seminiferous tubule in *Nsmce2* cKO testes compared to controls (Figures 4E,F). This indicates that NSMCE2 deficiency in germ cells does not cause widespread germ cell death. Finally, immunofluorescence staining for PCNA (a proliferating cell marker) revealed that the proliferation index of spermatogonia remained normal in the absence of NSMCE2 (Figures 4G,H). Collectively, analysis of these key cellular markers indicates that spermatogenesis proceeds following NSMCE2 deficiency with minimal impact on germ cell homeostasis, Sertoli cell presence, or overall testicular cytological architecture.

### Meiotic chromosome synapsis and DSB repair proceed normally following germ cell deficiency of *Nsmce2*

**FIGURE 5:**
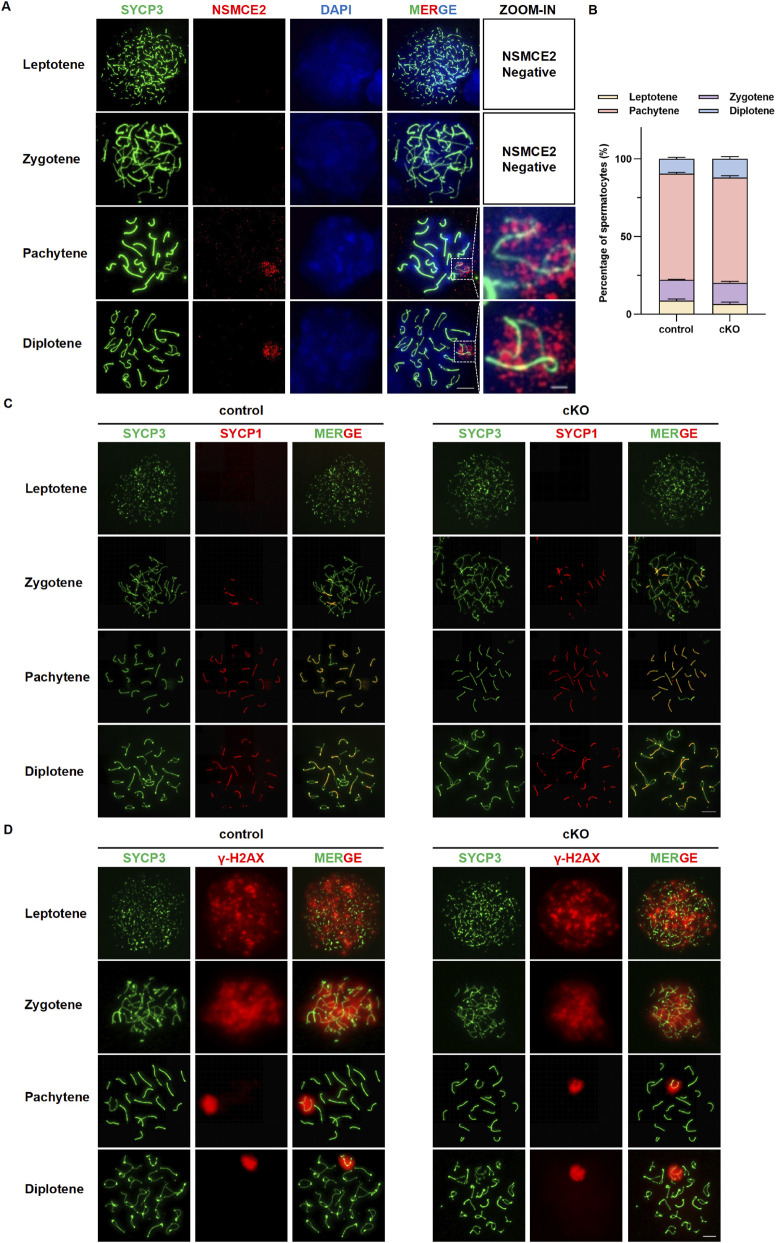
Meiotic chromosome synapsis and DSB repair proceed normally following germ cell deficiency of *Nsmce2* **(A)** Chromosome spreading and immunofluorescence staining in wild-type mice: SYCP3 (green), NSMCE2 (red), and DAPI (blue). Scale bar: 10 μm (main image); 2 μm (zoomed-in image) **(B)** The proportion of spermatocytes at different meiotic stages (leptotene, zygotene, pachytene, and diplotene) was quantified based on chromosome spreading. Data represent n = 10 biologically independent mice per group, with 100 spermatocytes analyzed per mouse. No significant difference between the two groups in any of the stages (*p* > 0.05) **(C)** Chromosome spreading and immunofluorescence staining in control and cKO mice: SYCP3 (green) and SYCP1 (red). Scale bar: 10 μm **(D)** Chromosome spreading and immunofluorescence staining in control and cKO mice: SYCP3 (green) and γ-H2AX (red). Scale bar: 10 μm.

Given the time-specific expression of NSMCE2 in testes and its known role in genomic stability, we next investigated its function during meiosis. Immunofluorescence analysis of chromosome spreads revealed the spatiotemporal localization of NSMCE2 in WT spermatocytes. During the leptotene and zygotene stages, NSMCE2 was undetectable on chromosomes. Strikingly, it was specifically enriched within the sex body throughout the pachytene and diplotene stages (Figure 5A). This unique localization pattern suggests that NSMCE2 may play a potential role in the sex body-specific chromatin environment during prophase I of meiosis.

**FIGURE 6:**
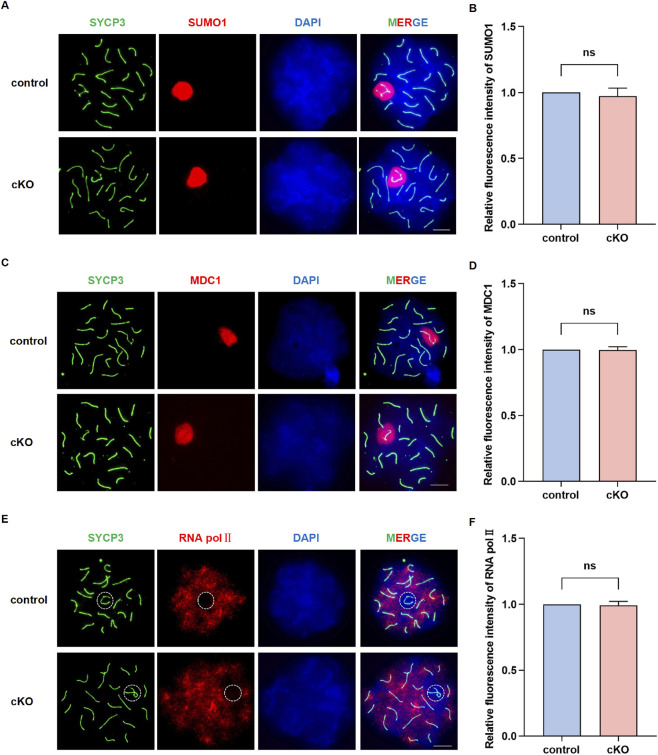
Normal establishment of meiotic sex chromosome inactivation (MSCI) in *Nsmce2*-deficient spermatocytes **(A,C,E)** Chromosome spreading and immunofluorescence staining in control and cKO mice: SYCP3 (green), SUMO1 (red) **(A)**, MDC1 (red) **(C)**, RNA Pol II (red) **(E)** and DAPI (blue). In **(E)**, the sex body are outlined by dashed circles. Scale bar: 10 μm **(B,D,F)** Quantitative analysis of fluorescence intensities of SUMO1 **(B)**, MDC1 **(D)**, RNA Pol II **(F)** on the sex body. Data represent n = 10 biologically independent mice per group, with 100 pachytene spermatocytes analyzed per mouse. ns, not significant.

Despite this specific localization, NSMCE2 deficiency in germ cells did not disrupt fundamental meiotic events. We first quantified the proportions of spermatocytes at different stages of prophase I. Our statistical analysis revealed that the proportion of leptotene, zygotene, pachytene, and diplotene cells in *Nsmce2* cKO mice was comparable to that in control groups, indicating that meiotic progression was not grossly impaired (Figure 5B). Consistent with this, staining of SYCP3, a lateral component of the synaptonemal complex, and SYCP1, a central component protein, revealed that homologous chromosome assembly and synapsis proceeded normally in *Nsmce2* cKO spermatocytes, indistinguishable from controls (Figure 5C). Furthermore, the repair of meiotic DNA double-strand breaks (DSBs), monitored by the formation and subsequent disappearance of γ-H2AX foci, remained unaffected. In control spermatocytes, γ-H2AX signaling was broadly present during the leptotene and zygotene stages and concentrated on the sex body during the pachytene and diplotene stages. This pattern was precisely reproduced in *Nsmce2* cKO spermatocytes (Figure 5D), indicating that signaling and repair of meiotic DSBs proceed efficiently in the deficiency of NSMCE2. To investigate whether *Nsmce2* deficiency compromises Meiotic Sex Chromosome Inactivation (MSCI), we examined the expression of key markers, including SUMO1, MDC1 and RNA Pol II (Figures 6A–F). During pachytene stage in cKO mice, SUMO1 and MDC1 were correctly localized to sex body, and RNA Pol II was effectively excluded from sex body, with fluorescence intensities of these markers all comparable to those in controls (*p* > 0.05). Collectively, these data demonstrate that *Nsmce2* is dispensable for the establishment and maintenance of MSCI.

In summary, although NSMCE2 exhibits a highly specific spatiotemporal localization pattern during meiotic prophase I, its deficiency in germ cells does not significantly impair chromosome synapsis, DSB repair or MSCI, possibly suggesting the existence of compensatory pathways that maintain meiotic integrity.

### Transcriptomic profiling revealed limited molecular disturbance in testes following germ cell deficiency of *Nsmce2*

**FIGURE 7:**
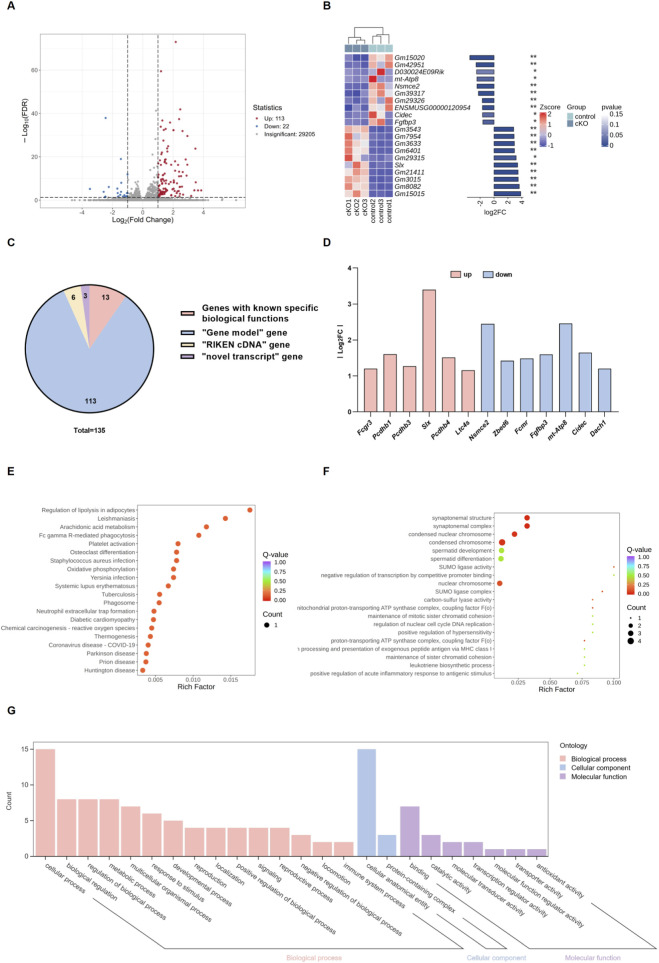
Transcriptomic profiling revealed limited molecular disturbance in testes following germ cell deficiency of *Nsmce2* **(A)** Volcano plot of the transcriptome from control and cKO testes **(B)** Heatmap of the top 10 most significantly upregulated and downregulated genes **(C)** Composition of the 135 differentially expressed genes (DEGs) **(D)** Expression levels (Log2 FoldChange) of the 13 annotated DEGs with known biological functions **(E)** KEGG pathway enrichment analysis of DEGs **(F)** GO classification statistics of DEGs **(G)** GO enrichment analysis of DEGs.

To determine whether *Nsmce2* loss induces transcriptional changes, RNA-sequencing was performed on adult testes from WT and cKO mice. Principal component analysis showed minimal separation between the two groups, indicating overall transcriptional stability (Figure 7A). Only a small set of genes exhibited differential expression (Figure 7B). Among these 135 altered genes, only 13 possess known biological functions (including *Nsmce2*). Genes related to meiosis, DNA repair, or spermatid differentiation remained largely unchanged (Figures 7C,D). Gene Ontology (GO) and KEGG analyses confirmed that only few differentially expressed genes (DEGs) were enriched in terms related to meiosis or chromatin maintenance. (Figures 7E–G). These findings suggest that the germline compensates for *Nsmce2* loss without widespread transcriptional remodeling, further reflecting the intrinsic robustness of the spermatogenic system.

### NSMCE2 deficiency reveals functional redundancy within the meiotic repair network

**FIGURE 8:**
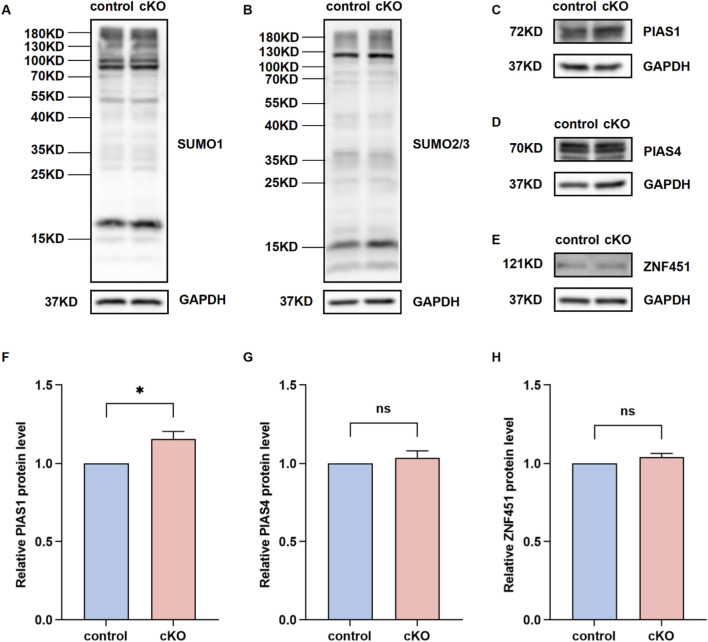
Compensatory upregulation of the SUMO E3 ligase PIAS1 in *Nsmce2*-deficient testes **(A,B)** Western blotting analysis of global SUMO1 **(A)** and SUMO2/3 **(B)** conjugates in testes from 8-week-old control and cKO mice (n = 3). GAPDH was used as an internal control **(C–E)** Western blotting analysis of the protein expression of other SUMO E3 ligases, including PIAS1 **(C)**, PIAS4 **(D)**, and ZNF451 **(E)** in testes from 8-week-old control and cKO mice (n = 3). GAPDH was used as an internal control **(F–H)** Quantitative analysis of the relative protein expression for PIAS1 **(F)**, PIAS4 **(G)**, and ZNF451 **(H)**. Data were normalized to GAPDH, and expressed relative to the control group (set as 1). *p < 0.05. ns, not significant.

Collectively, the absence of morphological, cytological, and transcriptional abnormalities in *Nsmce2* cKO testes suggests that meiotic progression is preserved through redundant or compensatory mechanisms. To investigate the molecular basis of this resilience, we examined the global SUMOylation landscape in mouse testes. Western blotting analysis revealed that the overall levels of SUMO1 and SUMO2/3 remained comparable between control and cKO groups (Figures 8A,B), indicating that *Nsmce2* deficiency does not compromise the global SUMO environment.

We hypothesized that other SUMO E3 ligases might functionally compensate for NSMCE2. We screened several candidate ligases known to operate in the germline, including PIAS1 (Constanzo et al., 2016; Shah et al., 2024), PIAS4 (Gallardo-Chamizo et al., 2025; Han et al., 2022), and ZNF451 (Bouchard et al., 2025; Cappadocia et al., 2015). While the protein levels of PIAS4 (Figures 8D,G) and ZNF451 (Figures 8E,H) remained unchanged, quantitative analysis revealed a significant upregulation of PIAS1 in cKO testes compared to controls (*p* < 0.05; Figures 8C,F). These findings suggest that PIAS1 may be upregulated to compensate for the deficiency of NSMCE2, potentially contributing to the maintenance of SUMO-dependent DNA repair and chromatin stability. Such potential functional redundancy of NSMCE2 likely supports the stability of the meiotic program under physiological conditions, highlighting the robust and fail-safe design of the male germline.

Thus, our findings reveal that *Nsmce2* is not essential for meiosis or fertility but instead exemplifies the system-level resilience of spermatogenesis, wherein multiple molecular pathways collectively safeguard genomic integrity.

## Discussion

In this study, we demonstrate that germ cell–specific deletion of *Nsmce2*, a SUMO E3 ligase subunit of the SMC5/6 complex, has no discernible impact on meiotic progression, spermatogenesis, or male fertility. Although NSMCE2 has been recognized as an essential factor for genome stability in somatic cells, its absence in the germline produced no overt phenotype. This finding, initially unexpected, provides valuable insight into the intrinsic robustness of the spermatogenic system. Rather than representing a negative result, it reveals how the germline is buffered against molecular perturbations through functional redundancy and compensatory mechanisms within the meiotic DNA repair network.

The SMC5/6 complex, together with cohesin (SMC1/3) and condensin (SMC2/4), constitutes a fundamental group of chromosome maintenance machineries that ensure faithful DNA replication, recombination, and segregation (Forni et al., 2024; Samejima et al., 2025; Ur and Corbett, 2021). Hwang et al. (Hwang et al., 2018) reported that the deficiency of the core scaffold subunit *Smc5* similarly resulted in fertile mice with no overt meiotic defects under physiological conditions. This phenotypic consistency between *Smc5* knockout and *Nsmce2* knockout in male germ cells provides robust evidence that the SMC5/6 complex is indeed dispensable for spontaneous meiotic recombination in mice. However, while Hwang et al. established the phenomenon of functional redundancy, the specific factors responsible for this compensation remained unidentified.

Our study fills this knowledge gap by proposing a potential mechanistic explanation. We observed that the chromatin environment of the male reproductive system is supported by compensatory mechanisms. Specifically, we found that the SUMO E3 ligase PIAS1 is upregulated in *Nsmce2*-deficient testes. This suggests PIAS1 may functionally substitute for NSMCE2, maintaining overall SUMOylation homeostasis and ensuring successful meiosis. By identifying PIAS1 upregulation as a potential compensatory mechanism, our work moves beyond phenomenological observations of non-essentiality to propose specific molecular mechanisms underlying this germline robustness. Notably, while Western blotting indicates unchanged global SUMOylation in cKO testes, we acknowledge that the bulk assay averages signals across the entire testis and may mask subtle fluctuations restricted to specific meiotic stages or subcellular domains. Furthermore, the functional complementarity between PIAS1 and NSMCE2 requires further investigation. We will establish *Nsmce2/Pias1* double-knockout model and *Pias1* knockout model in male germ cells to validate the underlying compensatory mechanism.

The concept of biological robustness—the ability of a system to maintain function in the face of internal or external perturbations—is well established in systems biology and developmental genetics (Osterwalder et al., 2018; Wen et al., 2025; Zhu et al., 2022). Spermatogenesis exemplifies such robustness: it integrates transcriptional (Geisinger et al., 2021; Rabbani et al., 2022), epigenetic (Andreu-Noguera et al., 2023; Kablan et al., 2025), and post-translational (Diawara and Martin, 2023; Lv et al., 2024) regulatory networks that collectively safeguard genomic integrity. The absence of overt defects in *Nsmce2*-deficient testes therefore reflects not a lack of importance, but rather the existence of a resilient molecular design in which individual nodes can fail without collapsing the entire network. This “fail-safe” architecture may have evolved to secure reproductive success, given the critical necessity of transmitting an intact genome to the next generation.

Our transcriptomic analyses further support this notion. Only minimal gene expression changes were detected in *Nsmce2* knockout testes, and meiotic or DNA repair genes remained stable. Such limited transcriptomic disturbance indicates that the spermatogenic system is able to preserve its global regulatory equilibrium, even when a key repair factor is removed. Interestingly, the few upregulated genes were associated with stress responses and cellular processes, implying a subtle compensatory activation of cellular defense pathways that buffer against molecular imbalance. Together, these findings highlight the dynamic homeostasis of the male germline—a system capable of self-correction and functional continuity despite perturbations.

It is also possible that the requirement for NSMCE2 is context-dependent rather than absolute. Under physiological conditions, alternative pathways may suffice to preserve meiotic recombination fidelity. However, under environmental or genotoxic stress—such as exposure to radiation, heavy metals, or oxidative agents—the absence of NSMCE2 might unmask latent vulnerabilities in the repair machinery. This context dependency aligns with the emerging view that gene essentiality varies with cellular state, tissue environment, and evolutionary context (Barchi et al., 2008; Chen et al., 2024; Homma et al., 2020). The findings of Hwang et al. strongly support this view: they discovered that *Smc5*-deficient mice exhibited enlarged round spermatids with supernumerary chromosome number following gamma irradiation and etoposide treatment. Therefore, systematically evaluating the impact of environmental stress on meiotic progression and fertility in germ cell–specific *Nsmce2* conditional knockout mice is the direction of our future research.

From an evolutionary perspective, functional redundancy is not merely a byproduct of gene duplication but a selected trait that enhances the resilience of critical biological processes. Meiosis, as a highly complex and error-sensitive event, may have evolved multilayered protection to prevent catastrophic failure. Redundant enzymes, overlapping DNA repair pathways, and compensatory chromatin remodelers form a distributed network that collectively preserves chromosomal integrity. Our findings therefore extend the understanding of meiotic control from a single-gene perspective to a system-level view of robustness, where stability emerges from the interplay of multiple, partially redundant modules.

To evaluate the clinical implications of our findings, we searched databases including ClinVar, Online Mendelian Inheritance in Man (OMIM), and the Male Fertility Gene Atlas (MFGA), but found no direct reports linking *NSMCE2* mutations specifically to human male infertility. Our findings of functional redundancy suggest that compensatory mechanisms, such as PIAS1 upregulation, might safeguard human fertility even in the presence of *NSMCE2* variants, potentially explaining the lack of infertility cases attributed to this gene.

In summary, the dispensability of NSMCE2 in mouse spermatogenesis provides conceptual evidence that the meiotic DNA repair network operates as a robust and redundant system. This redundancy ensures that essential reproductive processes remain functional even when individual components are lost. Beyond its specific implications for the SMC5/6 complex, our study exemplifies how null phenotypes can yield positive insights into the design principles of biological systems. Recognizing and characterizing such robustness is crucial for understanding reproductive biology, interpreting gene essentiality, and predicting the resilience of germline function under environmental challenges. Ultimately, the capacity of spermatogenesis to withstand genetic perturbation underscores an evolutionary truth: the continuity of life depends not only on genetic precision but also on the system’s ability to remain steadfast in the face of disruption.

## Data Availability

The datasets presented in this study can be found in online repositories. The names of the repository/repositories and accession number(s) can be found in the article/Supplementary Material.
