# Supplementary material for: NSMCE2 dispensability in mouse spermatogenesis suggests functional redundancy within the meiotic repair network
Source: Front Cell Dev Biol. 2026 Mar 3;14:1751806. doi: 10.3389/fcell.2026.1751806 (PMC12992291; doi:10.3389/fcell.2026.1751806)
Supplement: Supplementary file 1 [file Supplementaryfile1.docx]

**
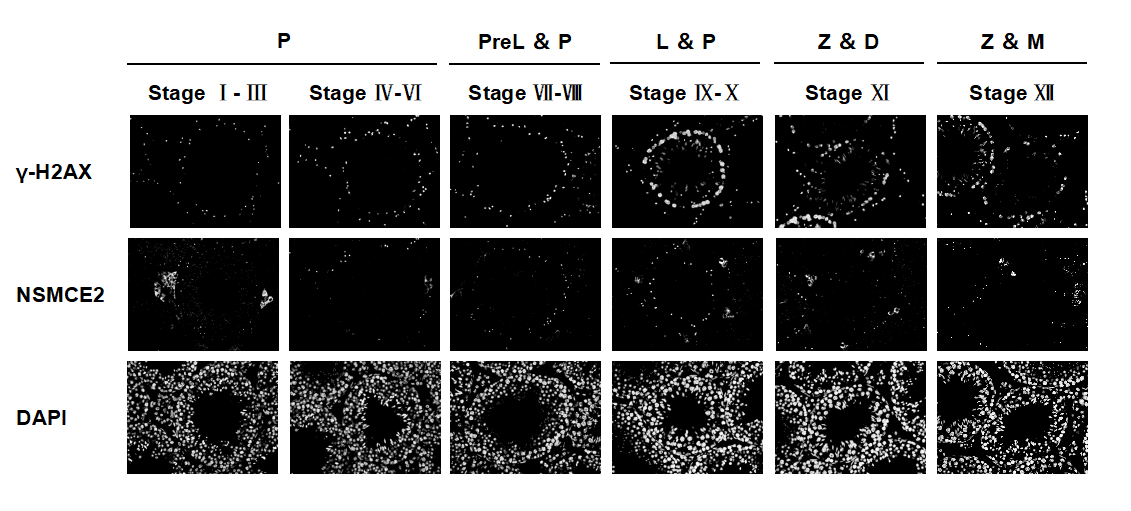
**

**Supplementary Figure 1.**

The black-and-white images for individual immunofluorescence channels of Figure 1F. Scale bar: 50 μm. P, pachytene; PreL, preleptotene; L, leptotene; Z, zygotene; D, diplotene; M, metaphase.


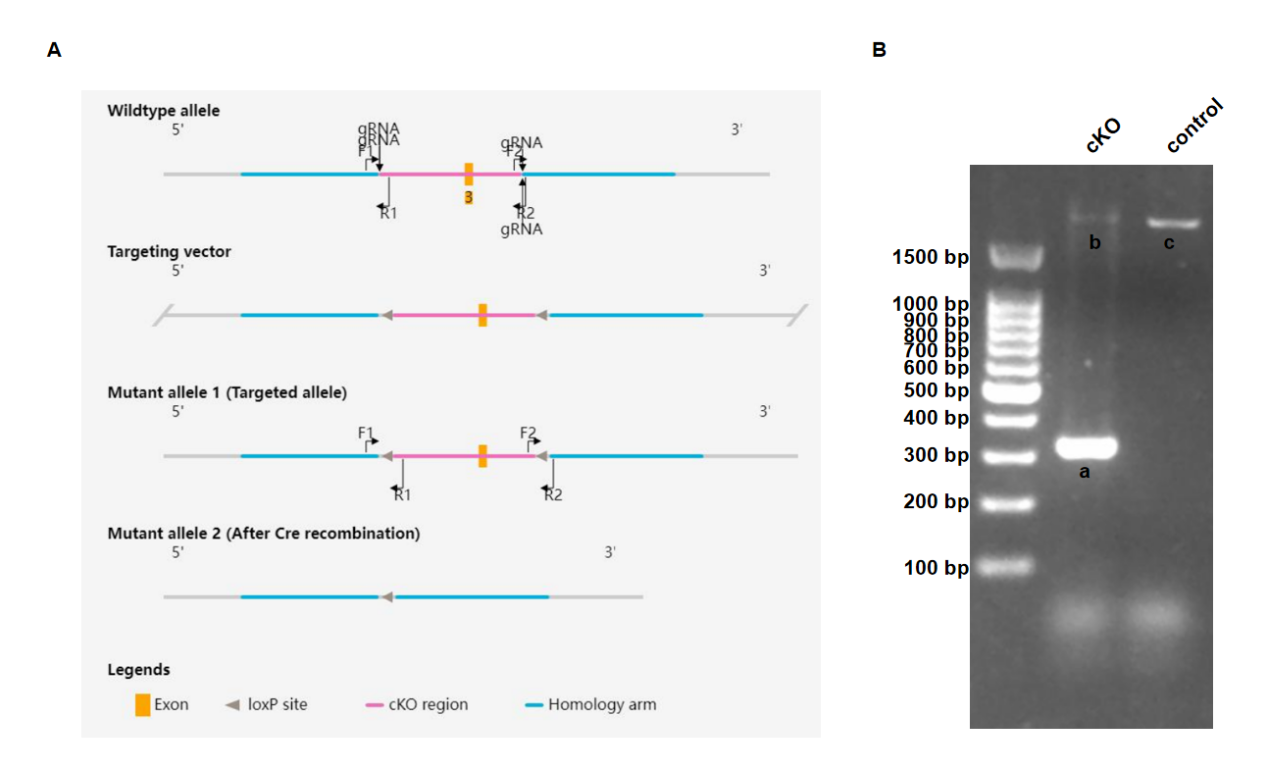


**Supplementary Figure 2. Molecular confirmation of the *Nsmce2* knockout band.**

1. Schematic diagram of the genotyping primer design strategy. (B) RT-PCR analysis of whole testis cDNA from control and cKO mice using primers F1/R2. Band “a”: predominant truncated transcript (about 300-400 bp) lacking the floxed exon, confirming Cre-mediated recombination. Band “b”: faint wild-type band (>1500 bp) originating from non-germline somatic cells (e.g., Sertoli cells) within the whole cKO testis lysate. Band “c”: expected full-length wild-type transcript (>1500 bp).
